# Ramadan fasting and exercise combination therapy: A novel approach for osteoporosis prevention in ovariectomized rats

**DOI:** 10.3389/fphys.2024.1403772

**Published:** 2024-10-23

**Authors:** Tarfa Albrahim, Raghad Alangry, Raghad Alotaibi, Leen Almandil, Sara Alburikan, Hisham S. Aloudah, Mohammed Alahmed, Mona Alonazi

**Affiliations:** ^1^ Department of Health Sciences, Clinical Nutrition, College of Health and Rehabilitation Sciences, Princess Nourah Bint Abdulrahman University, Riyadh, Saudi Arabia; ^2^ Prince Naif for Health Research Center, King Saud University, Riyadh, Saudi Arabia; ^3^ Research Office, Johns Hopkins Aramco Healthcare, Dhahran, Saudi Arabia; ^4^ Department of Biochemistry, College of Science, King Saud University, Riyadh, Saudi Arabia

**Keywords:** ovariectomy, Ramadan fasting, exercise, bone metabolism, estrogen deficiency

## Abstract

**Background:**

Osteoporosis is a chronic bone metabolic disease that affects millions of people worldwide, particularly the elderly and postmenopausal women. It is characterized by weakened bones, increasing the risk of fractures and leading to significant morbidity and mortality. The goal of the current study is to examine the reported osteo-preservative effects of exercise and/or fasting in the Ramadan fasting model (RFM) in ovariectomized (OVX) rats.

**Methods:**

The experimental intervention started 1 month following the ovariectomy procedure and consisted of five 15-min exercise sessions per week at 18–25 m/min and/or an approximately 13-h fast from sunrise to sunset (6:00 AM–19:00 PM). Serum bone metabolism biomarker levels were measured, and mineral concentrations in femoral ashed bones and digested serum were determined. Additionally, serum bone alkaline phosphatase (b-ALP), parathyroid hormone, osteocalcin, calcitonin, and vitamin D3 concentrations were measured using the competitive enzyme immunoassay technique.

**Results:**

Calcium, magnesium, and phosphorus showed a notable decrease in mineral concentration among OVX rat femurs compared with the combination group (OVX + RFM + E) and control groups. In addition, homeostasis of serum concentrations of calcium, magnesium, and phosphorus was observed to increase in the OVX + RFM + E group rather than in the OVX group without intervention when compared with a control group. Furthermore, fasting and exercise, either alone or concurrently with ovariectomy, induced a non-significant elevation in osteocalcin, parathyroid hormone, and vitamin D3, whereas b-ALP and calcitonin increased significantly compared with those in control rats.

**Conclusion:**

The combination of the Ramadan fasting model and moderate intensity exercises among OVX rats manifested advantageous effects in bone biomarkers compared with OVX rats without intervention. This could be recommended as a lifestyle modification that is protective against osteoporosis, especially in the context of depleted estrogen hormone after menopause.

## Introduction

Osteoporosis, a chronic bone metabolic disease characterized by weakened bones, which increases the risk of fractures, is a serious public health problem affecting millions of people, especially the elderly and postmenopausal women (Al-Ansari et al., 2022; Jaul and Barron, 2017). A bone fracture seriously affects the daily activities and quality of life of individuals and can result in complete paralysis or death (Al-Ansari et al., 2022). Worldwide, osteoporotic fracture occurs every 3 seconds, with over 8.9 million fractures occurring yearly (Jaul and Barron, 2017). The possible outcomes of vertebral compression fractures include pain, deformity, disability, and increased mortality (Al-Saleh et al., 2023). Furthermore, those with hip fractures experienced a significant increase in morbidity and medical expenses (Al-Saleh et al., 2023). Osteoporosis has many risk factors, such as disruptive endocrine changes like menopause, obesity, and vitamin D deficiency (Al-Ansari et al., 2022). Estrogen hormone deficiency may also lead to bone and muscle loss and impaired physical function, which leads to obesity, hyperleptinemia, and leptin resistance (Ezzat-Zadeh et al., 2017; Colleluori et al., 2022).

The prevalence of osteoporosis is noteworthy, even in regions like Saudi Arabia, where an epidemiologic analysis revealed that 34% of healthy women and 30.7% of men aged 50–79 years are osteoporotic (Ezzat-Zadeh et al., 2017). Given these statistics, screening recommendations suggest that women aged 65 years and older, or those between 60 and 64 years with additional risk factors, should undergo osteoporosis screening (Al-Ansari et al., 2022; Al-Saleh et al., 2023). Hence, early detection and intervention are crucial in mitigating the personal and societal costs associated with this condition.

A change in a lifestyle, such as quitting smoking, exercising, and following an appropriate diet, is one way to prevent osteoporosis (Alwahhabi, 2015). Exercise is considered to positively impact osteoporosis and bone mineral density (BMD) (Zhang et al., 2022). In this context, different types of physical exercise, including aerobic exercise, which speeds up the heart rate and breathing, strength exercise, balance exercise, high-impact exercise, resistance training, and plyometric exercise, which utilizes the stretch‐shortening cycle by using a lengthening movement (eccentric) which is quickly followed by a shortening movement (concentric), are used in clinical practice to maintain or increase BMD (Li et al., 2021). A study has reported that exercise can increase bone strength and functional performance in postmenopausal women (Watson et al., 2018). Recently, a study demonstrated an indirect effect of exercise on bone tissue, by reducing leptin levels and improving insulin sensitivity (Khalafi et al., 2023). Therefore, scientists began to study alternative diets such as fasting, which are thought to decrease the incidence of osteoporosis.

Intermittent fasting can increase bone mass by decreasing parathyroid hormones and osteoclastogenesis and enhancing the osteoblast mechanism in a rat model (Alrowaili et al., 2021). Ramadan fasting is a special type of fasting, where during fasting hours, eating, drinking, and smoking are not allowed, but individuals are allowed to eat or drink from sundown until sunset (Wang and Wu, 2022). Ramadan fasting hours range from 13 to 18 depending on the geographical area, as the fasting hours are more than intermittent fasting (Correia et al., 2020). The Ramadan model (RM) protects the body from pro-inflammatory factors including TNF-α and IL-6 (Correia et al., 2020). Moreover, a study found a significant reduction of lowering blood sugar and LDL after 1 month of RM (Jahrami et al., 2022). Several studies have suggested that there is an association between inflammatory factors and insulin resistance with increased bone resorption and, thus, osteoporosis (Sanguineti et al., 2014; Xia et al., 2012).

To date, the mechanisms behind how Ramadan fasting and exercise individually and in combination affect bone health in ovariectomized rats are not fully understood. Most previous studies have focused on either Ramadan fasting or exercise alone, and there is a lack of research examining the combined effects of Ramadan fasting and exercise on bone health. The primary objective of the current study is to observe the preventive approach of an exercise program combined with RFM on osteoporosis in ovariectomized rats. The aim of the study was to examine the preventive approach of Ramadan fasting model and exercise, individually and in combination, to determine the bone health on ovariectomy-induced osteoporosis in a rat model.

## Materials and methods

### Animal preparation

Fifty female Sprague Dawley rats aged 8 weeks (weighing approximately 300 ± 20 g) were housed in the experimental surgery and animal lab, faculty of medicine, King Saud University. All animals were kept under controlled environmental temperature (22°C ± 1°C), humidity (50%–55%), and 12 day/night cycle. All the experimental procedures followed the guidelines of the Institutional Animal Care and Use Committee (IACUC) of King Saud University.

### Ethical considerations

All the experimental procedures followed the guidelines of the Institutional Animal Care and Use Committee (IACUC) of Princess Nourah bint Abdulrahman University (approval no. HAP-01-R-059; IRB log number: 22-1141; category of approval: EXEMPT).

### Experiment design and grouping

All rats were randomly divided into five groups (ten rats in each group) as follows: 1) Control group, 2) OVX group, 3) OVX with RFM, 4) OVX with Exercise, and 5) OVX with both RFM and Exercise. The rats had free access to AIN 93G diet and filtered water. Nutritional markers, body weight (in grams), food intake (in grams), and water consumption (in ml) were measured weekly throughout the experiment period.

### Bilateral ovariectomy procedure

All animals were deeply anesthetized using flows (8% sevoflurane inhalation anesthesia and 3% oxygen) in a rat chamber. Rats were placed in the chamber until the deep anesthesia. The rat was placed on a surgical table (temperature was fixed at 37°C). After that, a sterilized fine surgical blade was used to cut a 1-cm opening at the center of the pelvis region skin, and the muscle was opened carefully. Then, both ovaries were pushed out gently using locking straight forceps fixed on the tube and the ligament. Then, the ovary was removed using a surgical cautery machine to avoid bleeding. Then, the muscle was closed with two sutures, and the same was done for the skin.

All the rats were placed in the recovery unit after the surgery under veterinary care until they healed. After that, all the animals were returned to their cages.

### Ramadan fasting model (RFM)

**FIGURE 1:**
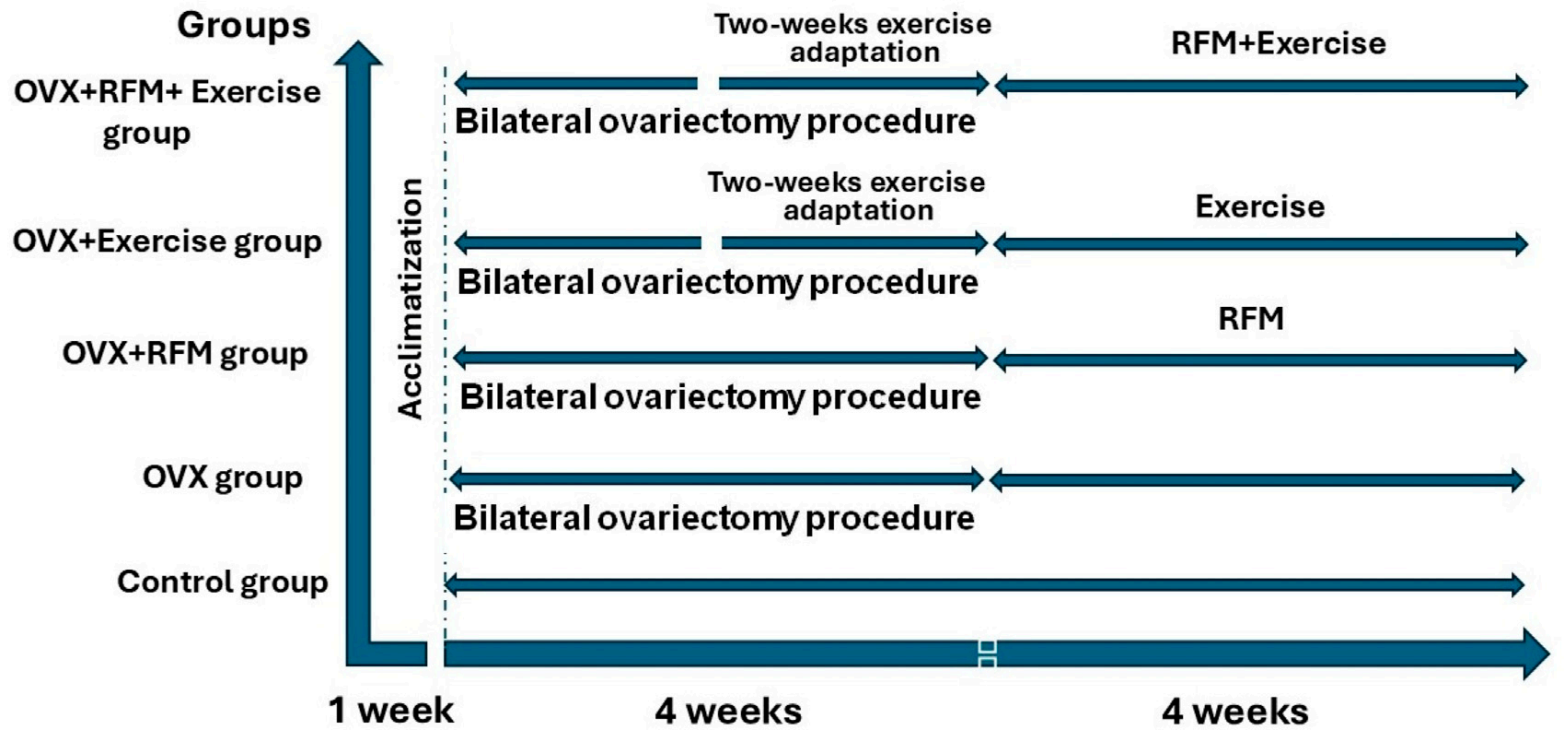
Diagram of schematic representation of the study protocol of the rat groups in weeks.

Postmenopausal female rats in the chronic phase after 1 month of bilateral ovariectomy were fasted from sunrise to sunset (6:00 AM–19:00 PM) according to Riyadh time, which estimates around 13 h. The fasting was forced by removing the food and water at dawn and returning them at sunset for 4 weeks. A schematic representation of the timing of different interventions is illustrated in Figure 1.

### Exercise session

After 2 weeks of bilateral ovariectomy, post-menopausal female rats in the chronic phase were trained on the treadmill for eight sessions over 2 weeks to adapt to the exercise. Then, animals were exposed to exercise five times a week on a 0% slope and for 15 min at 18–25 m/min. For 4 weeks, the running session lasted 30 min. A schematic representation of the timing of different interventions is illustrated in Figure 1.

### Sampling and sacrificing

All animals were anesthetized with ketamine/xylazine at a dose of (9.1/91 mg/kg); blood was withdrawn by using direct heart puncture and then transferred to a gel separation tube (yellow cap) and incubated at room temperature for 30 min before centrifuging at 3,500 RPM for 10 min, and the serum was kept at −80°C in a freezer until use.

The removal of muscle and tissue from the femoral bone started with washing the femoral bone. Next, the bone femoral was submerged in a solution of water and a gentle detergent. After soaking, the bone was removed from the solution and rinsed thoroughly with clean water. A sharp instrument was used to carefully scrape away any remaining tissue or mucus on the surface of the bone. Then, the bone was rinsed thoroughly with clean water once more to remove any remaining residue. The femoral bone was dried and stored in a cool, dry place until it was ready for use. After that, both sides of the femur were kept at the −80°C in a freezer until use.

Additionally, the kidney and liver were immediately removed and washed with isotonic saline. After the specimens were dissected, one half was promptly frozen at −80°C in order to harvest RNA. The second portion of the tissue was homogenized to a 10% (w/v) homogenate in 10-mM phosphate buffer (pH 7.4) that was ice-cold for biochemical investigations.

## Biomarkers

### Measurement of mineral concentration in femoral bone and serum

Dried femoral bones were weighed and placed in a heat-resistant pottery bowl at 700°C in a furnace for 16 h using muffle furnace (Lenton Thermal design, Hope Valley, United Kingdom). Then, the bone ash was collected and weighed to calculate the ash/bone ratio; moreover, 100 mg of bone ash was added to 3 µm of nitric acid and 0.5 µm of hydrochloric acid (HCl), mixed, and made to wait for approximately 10 min before closing the vessel for microwave digestion for approximately 20 min. For the serum, 0.5 mL of each sample was directly digested with 2 mL of nitric acid. The concentration of magnesium (Mg^+2^), phosphorus (P^+^), and calcium (Ca^+2^) were measured using inductively coupled plasma mass spectroscopy (ICP-MS) after being diluted with 45 mL of distal water to reach 70% of dilution and filtrated. The concentrations of minerals to ash in bones are reported as mg/100 bone ash, whereas mmol/L is used to express the concentrations in serum.

### Measurement of serum biochemical levels

The levels of serum bone alkaline phosphatase (b-ALP), parathyroid hormone (PTH), osteocalcin, calcitonin, and 25-hydroxyvitamin D3 (Vit D3) were then measured using rat-specific ELISA kits. ELISA kits with the following numbers were utilized: E-EL-R1109, E-PP-1730, E-EL-R0243, E-EL-R0047, and E-EL-0014, all from Elabscience in Wuhan, China.

### Assessment of the oxidative stress index

To determine the level of lipid peroxidation, malondialdehyde (MDA) was quantified using the thiobarbituric acid method developed by Ohkawa et al. (1979). As previously mentioned (Green et al., 1982), the amount of nitric oxide (NO) in the cerebral cortex was measured by measuring dye generation at 540 nm following the injection of Griess reagent. Ellman’s reagent was used to quantify the GSH levels, and the yellow chromogen was measured at 412 nm, as was previously described (Ellman, 1959). Furthermore, the manufacturer’s instructions were followed while using the Abcam (catalog number: ab65329; Cambridge, United Kingdom) colorimetric assay kit to measure the total antioxidant capacity (TAC).

Additionally, using the procedures described by Paglia and Valentine (1967), the activity of glutathione peroxidase (GPx) was assessed. The catalase (CAT) enzyme activity in the homogenates was determined using the method described by Aebi (1984). Superoxide dismutase (TSOD) activity was measured at 480 nm using the protocol described by Misra and Fridovich (1972).

### Measuring the inflammatory cytokines

The manufacturer’s instructions were followed while using the cyclooxygenase-2 (Cox-2), interleukin 1 beta (IL-1β), and tumor necrosis factor alpha (TNF-α) ELISA kits (MyBioSource, San Diego, CA, United States) to measure the levels of inflammatory cytokines. MBS725633, MBS2023030, and MBS175904, in that order, are the catalog numbers of the measured inflammatory markers.

### Real-time quantitative polymerase chain reaction

The harvested tissues’ total cellular RNA was separated with the aid of the TRIzol reagent (Invitrogen, Life Technologies Corporation, Carlsbad, CA, United States). We already mentioned the Q-PCR experiment and the reverse transcription reaction solution in our work (Albrahim et al., 2023). Using the 2^−ΔΔCt^ technique, the relative fold change was determined. The primers were synthesized by Sigma-Aldrich (St. Louis, MO, United States) and listed in Table 1.

**TABLE 1:** Gene primer sequences examined by real-time PCR analysis.

Name	Accession number	Forward primer (5'---3′)	Reverse primer (5'---3′)
GAPDH	NM_017008.4	CTC​TCT​GCT​CCT​CCC​TGT​TC	TAC​GGC​CAA​ATC​CGT​TCA​CA
SOD2	NM_017051.2	CGG​GGG​CCA​TAT​CAA​TCA​CA	GCC​TCC​AGC​AAC​TCT​CCT​TT
GPx1	NM_030826.4	CCT​GGT​ATC​TGG​GCT​TGG​TG	TTA​GGC​GTA​AAG​GCA​TCG​GG

### Statistical analysis

All data will be statistically analyzed using GraphPad Prism software version 9. The one-way ANOVA will assess the significant difference in minerals concentration between different groups, followed by Tukey’s *post hoc* test. Before using an analysis of variance (ANOVA), all parameters with continuous data were put through Levene’s test to ensure that the variance was homogeneous. A Mann–Whitney U test was used to determine the significance. The Shapiro–Wilk test was used to first determine whether the data were normal. The obtained data were displayed as the mean ± S.D. A p-value of equal or less than 0.05 is considered significant.

## Results

### Effects of exercise and fasting on body weight

**FIGURE 2:**
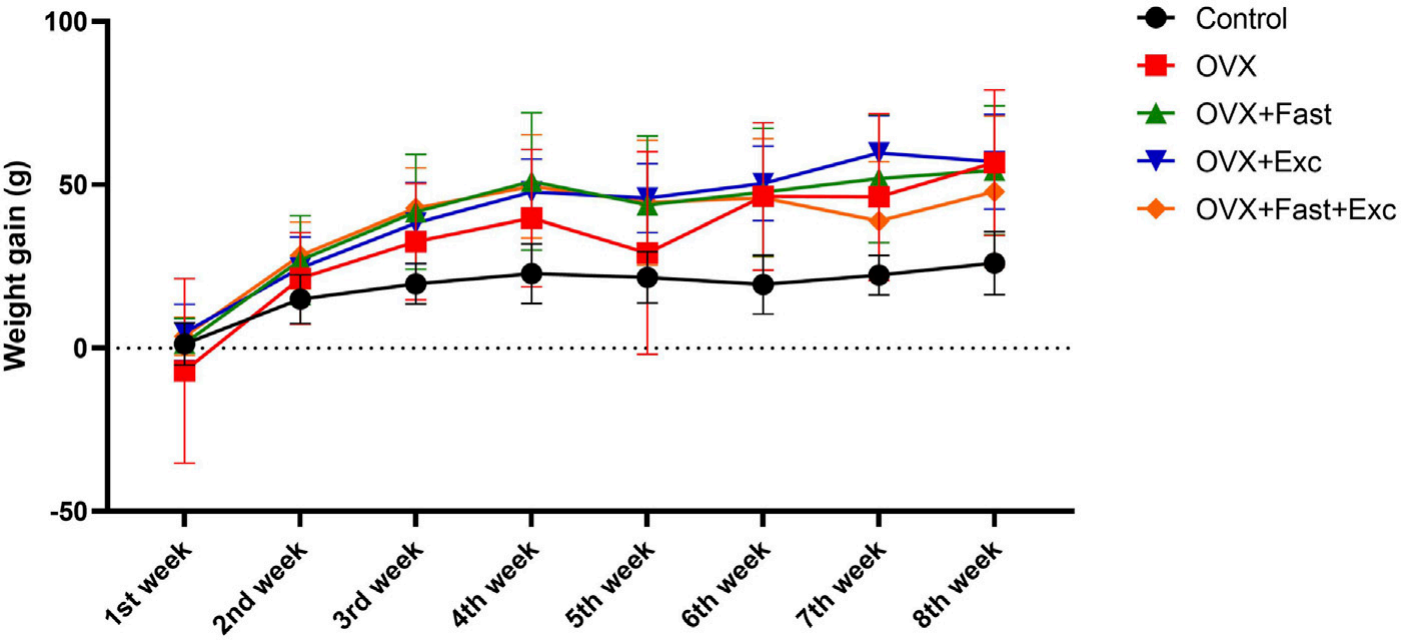
Effects of exercise and/or fasting on weekly weight gain.

Figure 2 displays the effects of exercise and fasting on weekly weight gain. At baseline, the body weights of all animals were within the same range (280–320 g). However, after 2 weeks of ovariectomy procedures, the gain in body weight was increased, as expected. There was no effect in the OVX, RFM, exercise, or combination of RFM and exercise groups to decrease the increment in body weight gain compared with the control group (p < 0.05).

The numerical parameters are presented as the mean ± S.D. of each group (n = 6/group).

### Weekly change in food and water consumption

**FIGURE 3:**
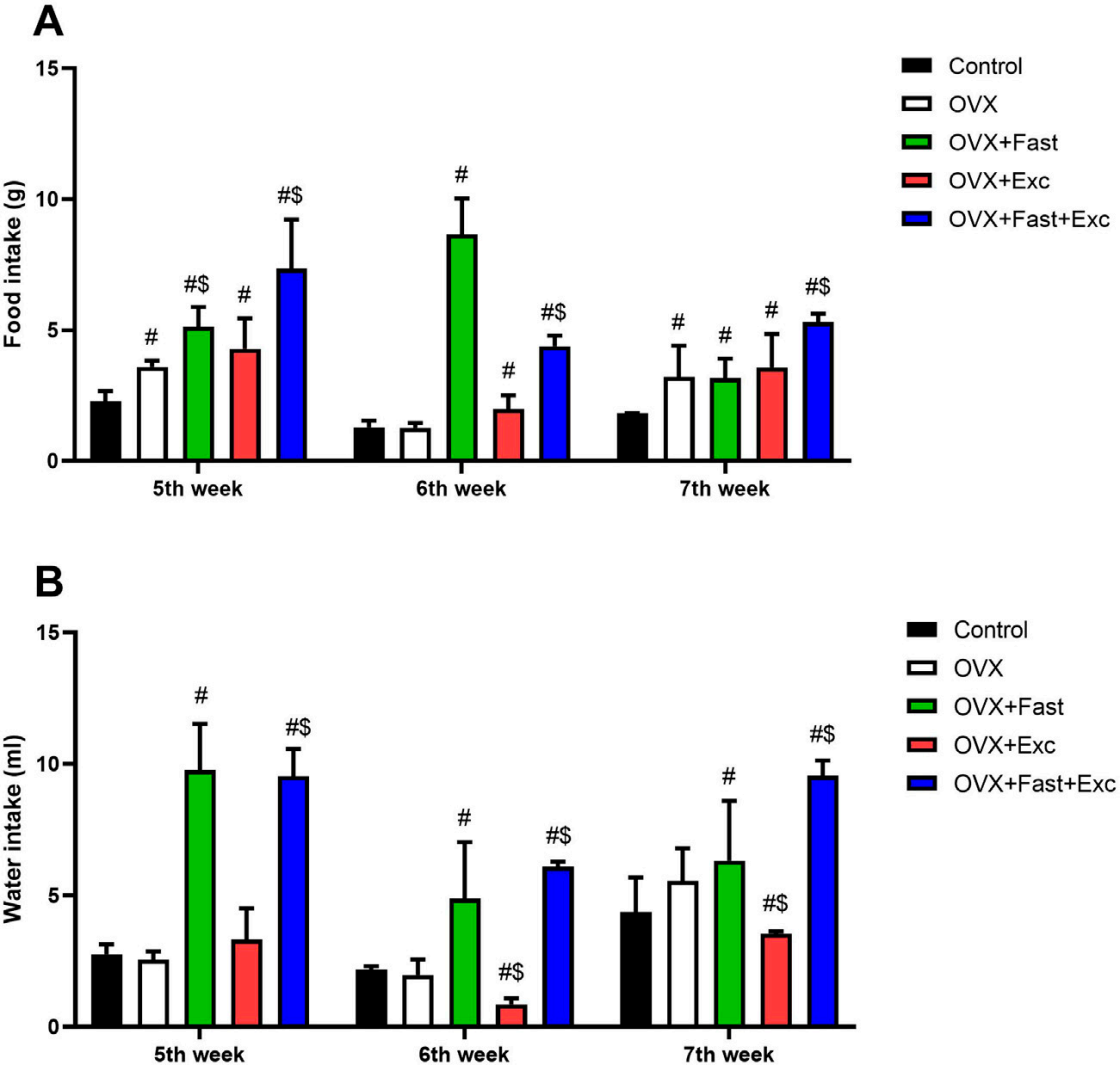
Change in **(A)** food consumption and **(B)** water consumption of all rats before intervention, after intervention, and at the end of the experiment. The numerical parameter is presented as the mean ± S.D. of each group (n = 6/group). Data were statistically evaluated using one-way ANOVA followed by the Tukey multiple comparison *post hoc* test. ^#^p < 0.05 compared to the control group; ^$^p < 0.05 compared to the OVX group.

Ovariectomized rats showed a significant increase in food intake before intervention and after ovariectomy procedures compared with the control rats (p < 0.05). However, at the end of the experiment, the combination of RFM and exercise significantly increased food consumption compared with that of control and ovariectomized rats (p < 0.05) (Figure 3A). Moreover, ovariectomy procedures in rats did not affect water intake (p > 0.05). After 1 week of intervention, RFM and combination of RFM and exercise significantly increased water intake compared with that in control and ovariectomized rats (p < 0.05). In addition, at the end of the experiment, there was a significant increase (p < 0.05) in water intake in the combination group (OVX + RFM + Exercise) compared with that in other groups (Control, OVX, OVX + Exercise, and OVX + RFM) (Figure 3B).

### Effects of exercise and fasting on bone minerals concentrations

**FIGURE 4:**
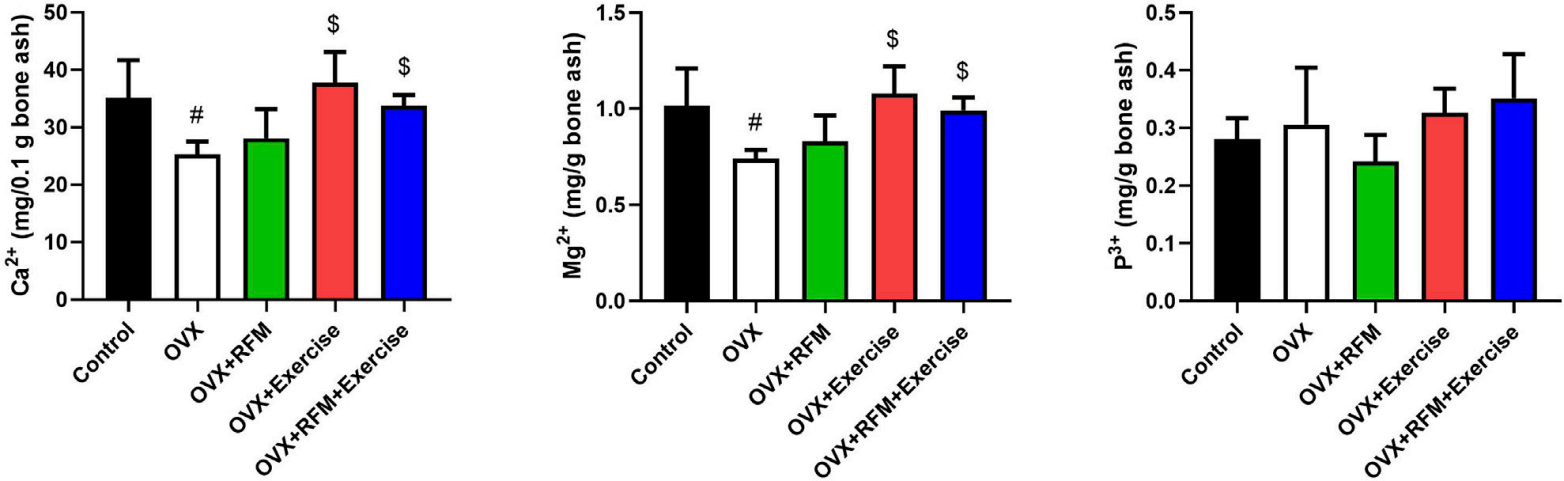
Effect of fasting and/or exercise on bone mineral concentration of femur bone in rats. The numerical parameter is presented as the mean ± S.D. of each group (n = 6/group). Data were statistically evaluated using one-way ANOVA followed by the Tukey multiple comparison *post hoc* test. ^#^p < 0.05 compared to the control group; ^$^p < 0.05 compared to the OVX group.

After 1 month of intervention, there was no significant difference in bone mineral concentration within the groups (p > 0.05). Otherwise, the OVX combination group (OVX + RFM + Exercise) and the OVX + Exercise group show approximately similar concentration in bone Ca^2+^ as the control group. In addition, non-intervention OVX groups show low bone Ca^2+^ compared to the OVX + Exercise group (Figure 4A). Moreover, the combination group (OVX + RFM + Exercise) had higher bone Mg^2+^ concentration than the control group (Figure 4B). Furthermore, the group that exercised over 1 month had more Mg^2+^ bone concentration than the OVX group without intervention. Interestingly, the combination group (OVX + RFM + Exercise) had more bone K^+^ concentrations than the control group. Otherwise, the OVX group had lower concentrations in bone K^+^ than the OVX + Exercise group (Figure 4C).

### Effects of exercise and fasting on serum minerals concentrations

**FIGURE 5:**
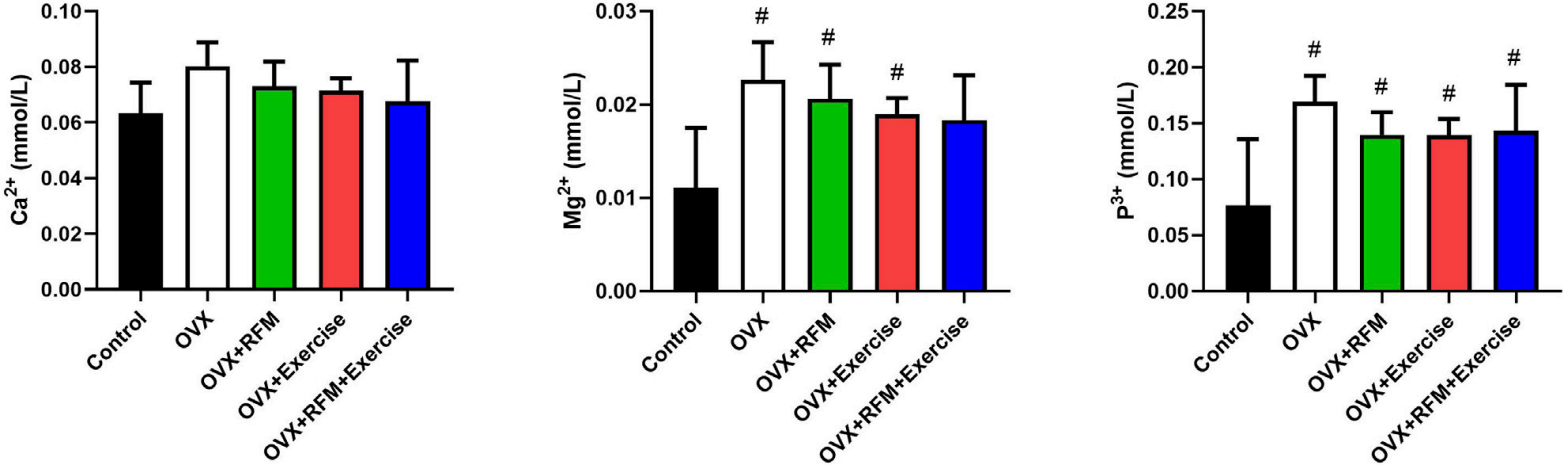
Effect of fasting and/or exercise on serum minerals concentration in rats. The numerical parameter is presented as the mean ± S.D. of each group (n = 6/group). Data were statistically evaluated using one-way ANOVA followed by the Tukey multiple comparison *post hoc* test. ^#^p < 0.05 compared to control the group; ^$^p < 0.05 compared to the OVX group.

The effects of exercise and fasting either alone or in combination on OVX rats are shown in Figure 5. At the end of the experiment, there is no significant difference in serum Ca^2+^ concentration within the groups (p > 0.05). However, the serum Ca^2+^ concentration in the OVX combination group (OVX + RFM + Exercise) is similar to that in the control group (Figure 5A). In addition, slightly positive effects on Ca^2+^ serum concentration were observed in OVX + RFM and OVX + Exercise groups compared with the OVX groups without intervention. Moreover, significant increase in serum Mg^2+^ concentrations was observed in the group that exercised along with the fasting combination intervention compared with that in the control group (p < 0.05) (Figure 5B). In addition, a significant increase (p < 0.05) was found in serum P^+^ concentrations in the ovariectomized rats groups (OVX, OVX + RFM, and OVX + Exercise) compared with the control group, and there was no notable change in serum P^+^ concentrations in the combination group (OVX + RFM + Exercise) when contrasted with the control group (p > 0.05) (Figure 5C).

### Effects of exercise and/or fasting on serum biochemical parameters

**FIGURE 6:**
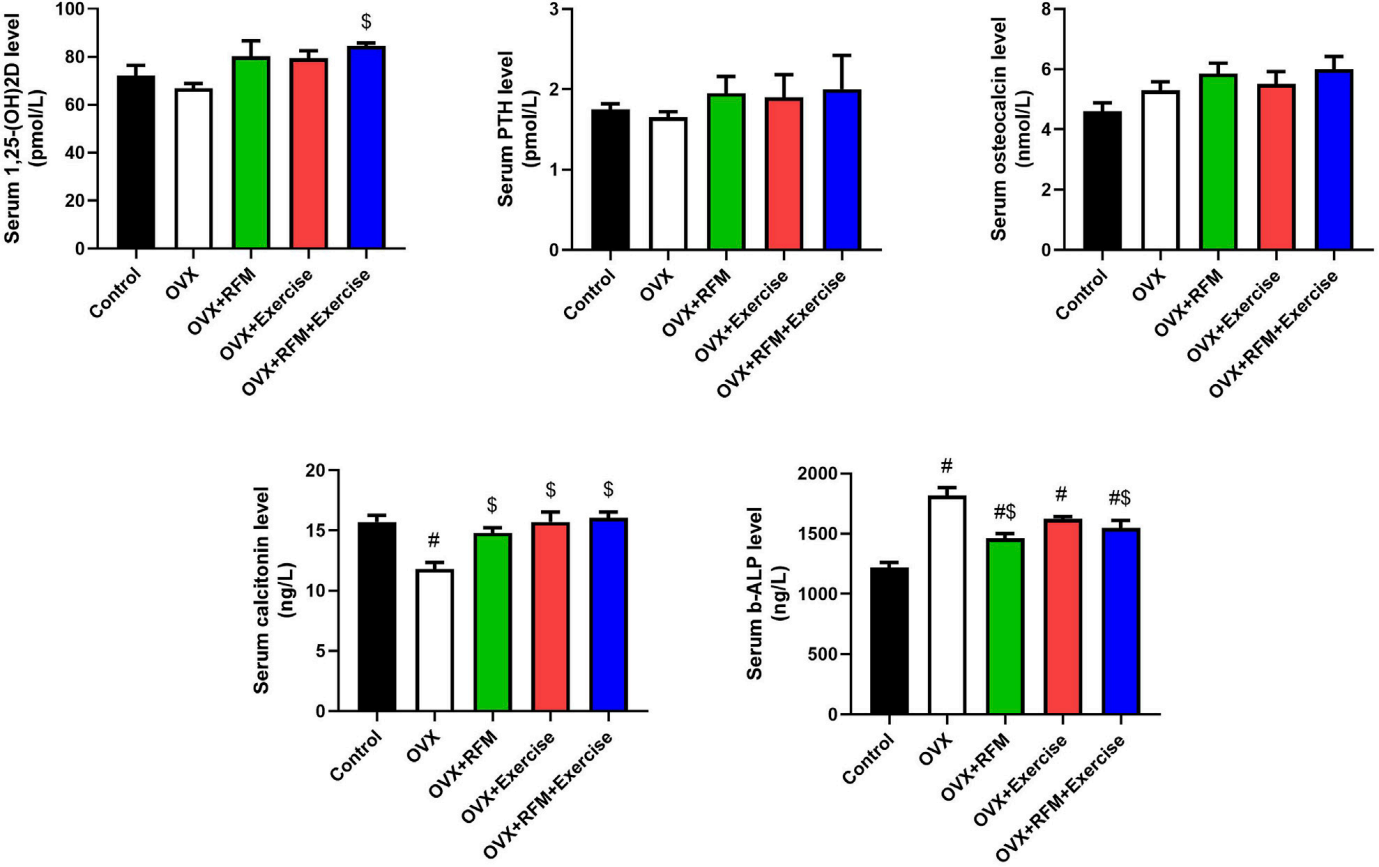
Effects of exercise and/or fasting on serum vitamin D3, osteocalcin, calcitonin, parathyroid hormone, and bone-alkaline phosphatase concentrations. The numerical parameter is presented as the mean ± S.D. of each group (n = 6/group). Data were statistically evaluated using one-way ANOVA followed by the Tukey multiple comparison *post hoc* test. ^#^p < 0.05 compared to the control group; ^$^p < 0.05 compared to the OVX group.

Ovariectomy in rat induced a non-significant decline in serum Vit. D3 compared to that in control rats. However, fasting and exercise, each one alone or concurrently with ovariectomy, induced a non-significant elevation in Vit. D3 compared with that in control rats. This increment in fasting and exercise, each one alone, was non-significant when compared with OVX rats, but the increment was significant during combined fasting and exercise with ovariectomy compared with that in OVX rats (Figure 6A). Furthermore, parathyroid hormone and osteocalcin levels were minimally decreased non-significantly in ovariectomy rats compared with that in control rats and increased non-significantly in fasting and exercise group rats, each one alone or concurrently with ovariectomy, compared to that in control and OVX rats (Figures 6B, C). However, the serum calcitonin level decreased significantly in ovariectomy rats compared with that in control rats. However, the decrease in calcitonin was non-significant in fasting and exercise group rats, each one alone or concurrently with ovariectomy, compared to that in control rats. Fasting and exercise, each one alone or concurrently with ovariectomy, induced significant increase in serum calcitonin compared to that in ovariectomized rats (Figure 6D). Additionally, the serum b-ALP level was increased significantly in ovariectomized rats compared to that in control rats. Moreover, exercise alone with ovariectomy failed to reduce the increment in b-ALP (Figure 6E). However, fasting alone or concurrently with exercise in ovariectomy rats significantly decreased the level of b-ALP compared to that in OVX rats, but the level was still significantly higher than that in control rats.

### Effects of exercise and/or fasting on tissue oxidative stress biomarkers

**FIGURE 7:**
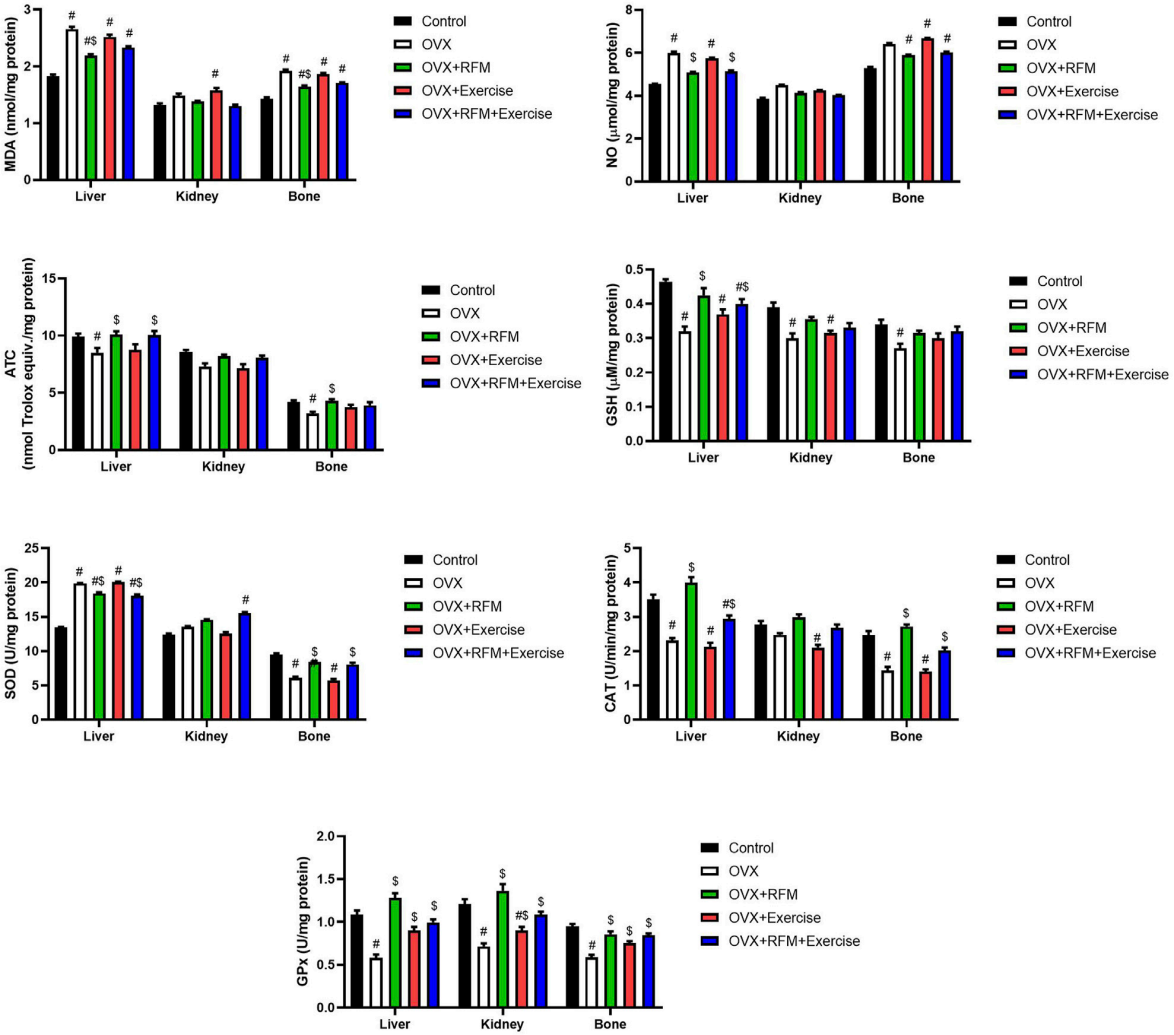
Effects of exercise and/or fasting on oxidative stress markers concentrations and mRNA expression of related genes in the liver, kidney, and serum. The numerical parameter is presented as the mean ± S.D. of each group (n = 6/group). Data were statistically evaluated using one-way ANOVA followed by the Tukey multiple comparison *post hoc* test. ^#^p < 0.05 compared to the control group; ^$^p < 0.05 compared to the OVX group.

**FIGURE 8:**
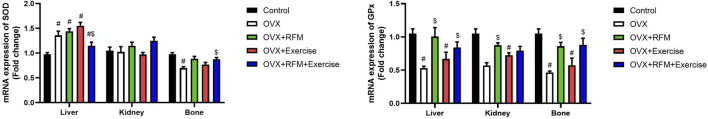
Effects of exercise and/or fasting on oxidative stress mRNA expression of related genes in the liver, kidney, and serum. The numerical parameter is presented as the mean ± S.D. of each group (n = 6/group). Data were statistically evaluated using one-way ANOVA followed by the Tukey multiple comparison *post hoc* test. ^#^p < 0.05 compared to the control group; ^$^p < 0.05 compared to the OVX group.

Ovariectomy in rats induced oxidative stress, evidenced by a significant increase in MDA (Figure 7A) and NO (Figure 7B) concurrently with a marked decrease in GSH (Figure 7C), associated with a significant inhibition in antioxidant enzymes, namely, SOD (Figure 7D), CAT (Figure 7E), and GPx (Figure 7F). Oxidants/antioxidants imbalance in ovariectomized rats’ liver, kidney, and bones was confirmed by the reduction in the total antioxidant capacity (TAS) level (Figure 7G). However, the obtained results revealed that ovariectomy is associated with a significant stimulation in SOD activity in the liver of rats. Interestingly, fasting of ovariectomized rats significantly attenuated oxidative stress induction. Exercise was not effective in the restoration of oxidants/antioxidants balance in ovariectomized rats. Furthermore, the combination of fasting and exercise also failed to restore the redox status in ovariectomized rats. mRNA expression of SOD2 (Figure 8A) and GPx1 (Figure 8B) confirmed the biochemical results, as indicated by significant downregulation in GPx1 and upregulation in SOD2 in the liver in ovariectomized rats, and fasting successfully attenuated this downregulation.

### Effects of exercise and/or fasting on tissue pro-inflammatory markers

**FIGURE 9:**
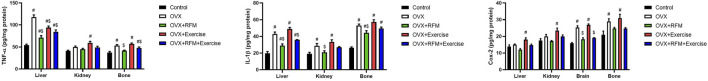
Effects of exercise and/or fasting on pro-inflammatory cytokines concentrations in the liver, kidney, and serum. The numerical parameter is presented as the mean ± S.D. of each group (n = 6/group). Data were statistically evaluated using one-way ANOVA followed by the Tukey multiple comparison *post hoc* test. ^#^p < 0.05 compared to the control group; ^$^p < 0.05 compared to the OVX group.

Ovariectomy significantly increased the pro-inflammatory cytokines/mediators compared to that in the control rats (p < 0.05). Exercise unfortunately augmented the inflammatory response in ovariectomized rats. However, fasting of the ovariectomized rats reduced the inflammatory response, evidenced by the significant reduction in TNF-α (Figure 9A), IL-1β (Figure 9B), and Cox-2 (Figure 9C). Moreover, the combination of fasting and exercise successfully restrained pro-inflammatory cytokines (TNF-α and IL-1β) and mediator (Cox-2) elevations.

## Discussion

Osteoporosis is a worldwide common disease that leads to many complications and increases morbidity and mortality, which causes a burden on health-care systems (Al-Ansari et al., 2022; Jaul and Barron, 2017). Lifestyle modification is the first line of treatment, and few studies investigated the effect of lifestyle on preventing osteoporosis. The ovariectomized rat model is one of the approved animal models to mimic postmenopausal, induced osteoporosis (Li et al., 2014; Yousefzadeh et al., 2020). In this sense, the current study investigated the protective effect of exercises and RFM and their combination against osteoporosis on the OVX rat model. Our results showed significantly increased body weight among the OVX group compared with the control group. These findings are explained by an observational cohort study; osteoporosis in postmenopausal women may be due to their altered hormonal status, including estrogen and follicle-stimulating hormone (FSH), which are noted to be associated with obesity occurring in women (Ebong et al., 2022). Apparently, the drop in circulating estradiol and the increase in FSH went along with the shift in visceral adiposity. Furthermore, menopause is linked to an increase in visceral and total body fat due to estrogen’s effects on regulating the expansion and metabolism of adipocytes, including lipolysis and lipoprotein lipase activity, as well as the correlated relationship between FSH and lipid production (Ebong et al., 2022; Curtis et al., 2018).

According to Burch et al. (2021), ovariectomized rats experience estrogen level depletion, which consequently leads to a rapid and significant increase in body weight, whereas intact rats gain weight slowly and more moderately. Moreover, the OVX group shows a significant increase in free fatty acids (FFAS) and triglyceride levels from baseline to complete 8 weeks. At the end of the experiment period, it was observed that the OVX group gained more weight than the control group (Curtis et al., 2018). Another experimental study carried out by Damirchi et al. (2010) made an interesting observation that ovariectomy in rats leads to a dramatic increase in body weight, as shown in the study results by the eighth week. Furthermore, there was a higher visceral fat level in the OVX group than in the control rats. These results of the previous studies are consistent with the findings of the present study. As was foreseeable, ovariectomy was followed by a significant gain in body weight after 2 weeks of the experiment. At the seventh week, there was a significant difference in body weight gain between the OVX and control groups. Furthermore, the weight gain continued in the OVX group until the end of the trial at the eighth week. A study demonstrated the effectiveness of exercise in postmenopausal women by reducing 6%–7% of body weight, leading to positive consequences on estradiol, free estradiol, androstenedione, and free testosterone as well as improved physical fitness (Moro et al., 2016). An additional study discovered that exercise dramatically decreased visceral fat in the OVX, with the exercise group contrasted to the OVX group; however, the body weight and BMI remained unchanged (van Gemert et al., 2015). In addition to exercise, fasting improves the metabolic state and weight loss and changes in body composition. A study conducted for 8 weeks demonstrated the effectiveness of fasting for 16 h/8 h feeding concluded that there was a drop in fat mass (Agostini et al., 2018).

Consistent with these studies, our findings observed that there was a positive effect of fasting and exercise on body weight change over the study duration, as observed when comparing the OVX–RFM group and the OVX–Exercise group with the OVX group. While previous studies’ findings are instructive in the context of exercise and fasting effects on body weight, the current study provides the effect of exercise and fasting combined on the body weight. There was a huge difference in body weight gain in the OVX, OVX + Exercise, and OVX + RFM groups with the control groups at the seventh week. However, by the eighth week of the experiment, it was noted that the control group alongside the combination group (OVX + RFM + Exercise) had the lowest body weight gains in contrast with the OVX, OVX + Exercise, and OVX + RFM groups. Overall, post-menopause leads to hormonal chaining linked to obesity in women. Moreover, fasting and exercise help promote losing body weight. Thereby, the combination of fasting with exercise shows a positive effect in terms of losing weight in the OVX rats. Our results support the hypothesis in accordance with the previous study, and this study shows significant increase in body weight and significant decrease in bone markers among ovariectomized rats without intervention. Previous studies illustrated a relation between sarcopenia and osteoporosis due to the biomechanical and biochemical relationship between muscles and long bones’ tissues (Barengolts et al., 1993). Skeletal muscle produces many proteins and peptides that can impact bone health; therefore, exercise is beneficial for osteoporosis prevention and treatment (Barengolts et al., 1993).

A study suggests that in the early stages of estrogen deficiency, endurance exercise can postpone the initial stage of bone mineral loss (Bahijri et al., 2015) and promote the development of muscle mass, endurance, and strength. Physical activity demands additional strain, so the formation of bone occurs, whereas lower strains encourage bone resorption; in addition, exercise can increase the myokines and osteokines, which are secreted by the muscle and bone, respectively, and has anabolic effects on the muscle and the osteoblast lineage through improving bone-forming cell differentiation and activity (Barengolts et al., 1993). However, it has been demonstrated that resistance exercise has a higher impact than endurance exercise on bone mass maintenance and improvement because of the nature of the mechanical stimulus. In addition, if we perform an endurance exercise without the right caloric intake, we can have the opposite effect with the induced protein catabolism. Amato et al. (2022) demonstrated the efficacy of resistance training in the elderly population on bone remodeling.

Furthermore, through changes in bone structure and/or localized adaptation in bone distribution in the regions subjected to the greatest strain, exercise training may improve bone strength independent of changes in BMD (Allison et al., 2015; Warden et al., 2007). A bone’s resistance to bending increases during exercise as a result of increased cortical thickness brought on by load-induced periosteal apposition and, to a lesser extent, decreased endocortical resorption (Warden et al., 2014). In this regard, Krogh et al. (2024) recently found that resistance training led to significantly increased levels of fasting N-terminal propeptide of type-I procollagen (P1NP, a bone formation marker) in both pediatric hematopoietic stem cell transplantation patients and controls with no significant changes in fasting C-terminal telopeptide of type-I collagen (CTX, a bone resorption marker) levels.

Dietary adjustments related to fasting during Ramadan regulated PTH secretion in a way that would be advantageous to bone health (Adawi et al., 2017). Fasting may have a positive impact on bone turnover while also lowering pro-inflammatory markers. A shift in eating habits during RFM influences PTH secretion by raising the calcium content in the evening and reducing bone resorption at night (Adawi et al., 2017; Hisatomi and Kugino, 2019). Furthermore, the current study discovered that intermittent fasting for 16–18 h in rats led to a large rise in serum levels of osteoprotegerin and a significant drop in serum levels of RANK, indicating that RFM can suppress osteoclast activity and stimulate osteoblast activity (Park and Shin, 2022). Similarly, Alrowaili et al. (2021) found that in rats with glucocorticoid-induced osteoporosis that were subjected to intermittent fasting for 16–18 h per day for 90 days, the serum levels of the bone formation biomarkers, namely, osteoprotegerin, ALP, and osteocalcin, were significantly increased, and those of the bone resorption markers, namely, tartrate-resistant acid phosphatase (TRAP)-5b, amino-terminal cross-linking telopeptide of type-I collagen (NTX-1), and deoxypyridinoline (DPD), were significantly decreased. This suggests that intermittent fasting slows the progression of glucocorticoid-induced osteoporosis by inhibiting osteoclast activity and promoting osteoblast osteogenesis. In a similar vein, it was discovered that intermittent fasting counteracted both the rise in the serum bone resorption marker TRAP and the BMD reduction brought on by a ketogenic diet (Xu et al., 2019). Controversy around animal studies shows that fasting shows no significant differences in lumbar vertebral body height and cortical bone thickness compared to the non-fasting group; however, the duration of fasting in the study was only 4 days (Masanova et al., 2022).

Supporting the beneficial effect of fasting, in a 6-month randomized controlled trial, the effects of alternate-day fasting regimens and caloric restriction on bone metabolic markers in overweight and obese individuals were investigated in relation to religious Ramadan fasting. The results showed good weight reduction in both the caloric restriction and alternate-day fasting groups, and no significant effects on bone mineral content (BMC), bone mineral density (BMD), or the markers related to bone metabolism, type-I collagen carboxy-terminal peptide (CTX-1), and OPG (Barnosky et al., 2017) were observed. Comparable findings were reported by Clayton et al. (2020), who discovered no impact of a 24-h fast on serum levels of PTH, procollagen type-I N-terminal propeptide (PINP), or CTX-1. In addition, Martens et al. (2020) demonstrated that neither the total BMD nor the regional BMD of middle-aged and elderly non-obese individuals differed from a control group after 6 weeks of time-restricted feeding, nor did these individuals’ bone mass decrease.

In clinical studies, Ramadan fasting could relieve the symptoms of the patients with RA or SpA by regulating inflammatory cytokines (decreasing CRP, IL1, or 6), lipid profiles, antropometric features, or intestinal microbial compositions (Ben Nessib et al., 2021; Rodopaios et al., 2024). As RIF has been found to have a beneficial effect on the secretion of PTH, it was hypothesized that it could positively affect the bone metabolism. RIF has also a positive impact on the activation of dipeptidyl peptidase 4 (DPP-4) inhibitors as the DPP-4 gene has been identified as an important genetic factor contributing to the progression of osteoporosis (Ben Nessib et al., 2021; Bahijri et al., 2015). However, there are no clinical investigations and basic research that indicates that RIF and exercise can ameliorate osteoporosis.

Overall, there is a significant reduction in bone density among ovariectomized rats, especially in the concentrations of calcium and magnesium in the bones, and this is mainly due to the depletion in the estrogen level (Lei et al., 2009; Vaananen and Harkonen, 1996). Estrogen deficiency affects bone density by increasing PTH, IL-1β, and IL-6, which are cytokines that increase the process of osteoclasts formation (Stern et al., 1988; Masanova et al., 2022).

The current study results found that the femur bone calcium concentration and serum calcium concentrations were higher in the OVX + Exercise group than in the OVX group without interventions. Recently, in some studies, the relationship between calcium homeostasis and exercise had been investigated. Some experimental animal studies found that there is an increase in the absorption of calcium from the intestine due to the stimulation of exercise to increase bone density, which leads to an increase in vitamin D3 absorption, resulting in the inhibition of PTH secretion (Yeh and Aloia, 1990; Ashizawa et al., 1997). Furthermore, exercise can help balance calcium levels in the bones and blood (Tella and Gallagher, 2014). Calcium homeostasis is important because a chronical decrease in calcium serum concentrations is associated with increase in PTH levels, which leads to the replacement of calcium from the bones to the blood (Peterson and Heffernan, 2008).

The results of the current study also showed that there was a slight unremarkable elevation and hemostasis in the OVX + RFM group compared to the OVX group in the serum samples. To explain our results, a study by Park and Shin (2022) found that intermittent fasting protects bone mass by reducing TNF-α levels in the serum. TNF-α can affect calcium absorption by decreasing the level of calcitriol in the blood (Attarzadeh Hosseini et al., 2013). In contrast, previous studies that examined the effect of Ramadan fasting on blood calcium levels have shown that blood calcium levels may be normal or slightly low due to the increased excretion of calcium in the urine and due to PTH abnormalities after Ramadan fasting (Bahijri et al., 2015; Castiglioni et al., 2013).

The main results of the current study investigated the effect of exercise and fasting on OVX rats compared with the control group. There is an identical result of calcium concentration in the bones and serum of the OVX + RFM + Exercise group compared to that in the control group that had not undergone an ovariectomy surgery, and this may be due to the combined benefits of fasting and exercise in regulating PTH and RANKL levels.

In addition, magnesium deficiency affects the absorption of vitamin D and PTH, which affects the absorption of calcium and bone density (Vazquez-Lorente et al., 2020; Marcus, 2018). Furthermore Vazquez-Lorente et al. (2020) investigated the mechanism effect of magnesium in bones, and they found that hypomagnesemia was associated with a increase in cytokines that accelerate the process of osteoclast, resulting in an increase in bone stiffness and osteoclast’s function. In OVX rats, they were exposed to magnesium deficiency, as depletion of the estrogen level was associated with low magnesium and BMD due to an increase in pro-inflammatory factors (Toba et al., 2000). In the experimental animal study, OVX rats fed with magnesium supplements had shown an increase in osteocalcin, which is a protein matrix that is considered as a marker for bone building. Our findings demonstrated higher Mg^2+^ bone concentration in the OVX + Exercise group than in the OVX group. Previous experimental animal studies have shown that there is a strong relationship between aerobic exercise such as treadmill and swimming and an increase in BMD by stimulating bone formation processes (Kang et al., 2017; Oh et al., 2016), which includes magnesium concentrations in the bones.

In our main finding, there was a notable increase in the bone magnesium level and serum magnesium level in comparison between the combination groups (OVX + RFM + Exercise) versus the control group, which supports the effectiveness of intervention in homeostasis magnesium concentration.

Our experimental results showed that ovariectomy in rats leads to a decrease the in bone potassium concentration level compared with that in the control group, potentially contributing to the development of osteoporosis. This finding is consistent with previous experimental animal studies conducted by Lei et al. (2009) and Ha et al. (2020), which reported a significant decrease in bone potassium concentrations levels in ovariectomized rats. Moreover, the present study found that OVX + Exercise group and OVX + RFM group interventions have differential effects on bone potassium concentrations in ovariectomized rats compared with the OVX group without intervention. In addition, the combination group (OVX + RFM + Exercise) had a higher bone potassium concentration level than the control group, which indicated an increase in BMD and a protective approach against osteoporosis (Ji et al., 2007).

Our findings also show a significant increase in serum potassium concentrations in the ovariectomized groups compared to that in the control group, consistent with a previous experimental study conduct by Kaastad et al. (2001), which claimed that the decrease in potassium concentrations levels in OVX rats is due to the deficiency of estrogen hormone, which causes the deficiency of the aldosterone hormone level.

Furthermore, an experimental animal study found that ovariectomy led to changes in metabolic function, specifically an increase in blood glucose levels and a decrease in insulin sensitivity. These findings suggest that the observed increase in potassium levels in the current study may be due to changes in metabolic function caused by ovariectomy (Shaban et al., 2017). Another possible explanation by Ciarambino et al. (2022) is that the increase in potassium levels in the ovariectomy group may be related to changes in renal function. Potassium levels are primarily regulated by the kidneys, and ovariectomy has been shown to alter renal function in rats, specifically a decrease in renal blood flow and the glomerular filtration rate.

In our findings, there was no significant increase or decrease in serum potassium levels in the combination group (OVX + RFM + Exercise) compared with that in the control group, suggesting that the combination of exercises and fasting may have improved effect in potassium homeostasis.

Vitamin D affects bone density, especially in the femur bones, and this effect increases when estrogen and vitamin D deficiency occur in cooperation, which leads to the high risk of fractures (Lips and van Schoor, 2011; Ali et al., 2014). This is the reason how vitamin D supplementation can prevent osteoporosis in postmenopausal women (Elwakeel et al., 2020). Vitamin D deficiency can cause imbalance in BMD levels, resulting in potassium and calcium deficiency (Lips and van Schoor, 2011). Furthermore, vitamin D suppresses the parathyroid hormone, which leads to increased calcium absorption in the intestine and deposition in the bones (Lips and van Schoor, 2011; Elwakeel et al., 2020). Our results reflect a decrease in serum levels of vitamin D in OVX rats. The relationship between OVX rats and depletion of the vitamin D level is explainable by the study of Sundell and Björnsson, who found a significant increase in the level of vitamin D in the serum of OVX rats after treatment with estrogen supplements (Sundell and Bjornsson, 1990). In another experimental animal study, there was a positive correlation between estrogen and vitamin in rats, as vitamin D increases estrogen and *vice versa* (Nashold et al., 2009). This process is due to the enhancement of the estrogen hormone of the vitamin D receptors in the central nervous system, which increases the absorption of vitamin D in the blood (Dzik et al., 2022).

Our study suggested that exercise intervention in the OVX + Exercise group can increase the level of vitamin D serum when compared with that in the OVX group. In agreement with our results, a randomized control trail found a significant increase in the vitamin D level in the participant after aerobic exercise (Dzik et al., 2022). In another experimental animal study conducted by Aly et al. (2016), they found a positive effect of swimming exercise on vitamin D serum among albino rats. This action may be due to the ability of exercise to increase the anabolic reaction of vitamin D in various tissues, especially in the muscle (Aly et al., 2016).

A recent study by Karras et al. (2023) investigated the effectiveness of intermittent fasting on vitamin D concentration among overweight participants. Interestingly, free 25-hydroxy vitamin D [25(OH)D] in the fasting group showed an increase that was considerable for the full study duration. In contrast to the control group, the fasting group demonstrated increased free 25(OH)D concentrations at the seventh week. Moreover, it was observed that body fat has an inverse relationship with vitamin D (Karras et al., 2023). This finding supports the present study results, which show an increase in serum vitamin D levels among the fasting group compared with the control group.

In the present study, it was found that the combination effect of RFM and exercise interventions in OVX rats eliminate the negative effects of ovariectomy and estrogen deficiency on decreasing the serum vitamin D level when compared with that in the control group.

However, in the present study, one possible explanation for the lack of effect of ovariectomy on calcium and vitamin D levels could be related to the timing of the measurements. It is possible that changes in calcium and vitamin D levels as a result of ovariectomy take longer than 1 month to manifest (Lei et al., 2009). Additionally, it is important to consider additional measures of bone health in conjunction with calcium and vitamin D levels, such as bone mineral density or bone microarchitecture. These measures may provide a more comprehensive understanding of the effects of ovariectomy on bone health in rats.

Overall, our results support the hypothesis accordant with the previous study, and this study shows significant increase in body weight and decrease in bone markers among ovariectomized rats without intervention.

### Study limitations

The study by Alrowaili et al. (2021) used an 18-h IF protocol for 3 months, whereas the current work only covered 8 weeks. This shorter duration may be insufficient to observe the full effects of IF on bone metabolism and osteoporosis management. In addition, our work may not fully account for the potential impact of nutritional changes during IF, particularly protein intake, which can significantly affect bone metabolism. Moreover, further study is needed for more detailed cellular investigations of bone cells to better understand the mechanisms mediating osteoporosis in this context. The current study may not provide sufficient insight into the cellular processes involved.

## Conclusion

In conclusion, this study demonstrated that a combination of exercise and Ramadan fasting intervention can regulate calcium, magnesium, and potassium homeostasis in femoral bone and serum concentration, in addition to maintaining bone metabolism, oxidative stress, and inflammatory status levels in the liver, kidney, and bone, which have a positive effect on bone health. The combination of the Ramadan fasting model and moderate intensity exercises could be recommended as a lifestyle modification that is protective against osteoporosis, especially in the context of depleted estrogen hormone after menopause. However, further investigation is needed to confirm the results of this study.

## Data Availability

The raw data supporting the conclusions of this article will be made available by the authors, without undue reservation.
